# Serum YKL-40, but Not Relaxin-2, Shows Diagnostic Utility as an Adjunct Biomarker in Colorectal Cancer

**DOI:** 10.3390/ijms262311601

**Published:** 2025-11-29

**Authors:** Kamil Safiejko, Marcin Juchimiuk, Julia Doroszkiewicz, Barbara Mroczko, Monika Zajkowska

**Affiliations:** 1 Department of Oncological Surgery with Specialized Cancer Treatment Units, Maria Sklodowska-Curie Oncology Center, 15-027 Bialystok, Poland; kamil.safiejko@gmail.com (K.S.); jumedica.onkologia@gmail.com (M.J.); 2 Department of Biochemical Diagnostics, Medical University of Bialystok Clinical Hospital, 15-269 Bialystok, Poland; barbara.mroczko@umb.edu.pl (B.M.); monika.zajkowska@umb.edu.pl (M.Z.); 3 Department of Neurodegeneration Diagnostics, Medical University of Bialystok, 15-269 Bialystok, Poland

**Keywords:** CHI3L1, RLN2, biomarker discovery, diagnostic panel, serum diagnostics

## Abstract

Despite the availability of conventional serum markers for colorectal cancer (CEA, CA 19-9), there remains a need for more sensitive and specific biomarkers, particularly for early-stage detection. This study evaluated the diagnostic usefulness of serum Relaxin-2 (RLN2) and Chitinase-3-like protein 1 (YKL-40) as potential adjunct markers in patients with CRC. Serum concentrations of all the proteins were measured using a multiplexing assay and CMIA and were subsequently compared using non-parametric statistical tests. The concentrations of YKL-40, CEA, and CA 19-9 were elevated in CRC patients relative to controls (*p* < 0.05), but not so for RLN2. The concentrations of YKL-40 were also significantly elevated in patients undergoing chemotherapy or preoperative radiotherapy referral. Kruskal–Wallis and post-hoc testing found that YKL-40 and CEA were associated with tumor progression, but RLN2 and CA 19-9 were increased primarily in advanced, metastatic disease. No statistically significant differences in marker levels were observed between cancer subtypes or between histologic grades. Performance analysis for diagnostic purposes showed YKL-40 was moderately sensitive (65%) but very specific (77.5%), and its AUC was 0.702, higher than CA 19-9 (AUC = 0.632) but lower than CEA (AUC = 0.869) (all *p* < 0.05). RLN2 did not reach statistical significance (AUC = 0.593, *p* = 0.09). Correlation analysis demonstrated the best correlation with disease stage for CEA and weaker positive correlations for YKL-40, CA 19-9, and RLN2. These findings suggest that YKL-40 may serve as a useful adjunct serum biomarker for CRC diagnosis, especially when combined with conventional markers such as CEA.

## 1. Introduction

Colorectal cancer (CRC) ranks as the third most frequently diagnosed malignancy and the second leading cause of cancer-related mortality globally, accounting for approximately two million new cases and more than 900,000 deaths in 2022 [1]. The weighted mean age-specific rates (ASR) are 18.4 and 8.1 per 100,000, respectively. Approximately half of all new colorectal cancer cases occurred in nations with a relatively high Human Development Index (HDI). Premature death rates in high-HDI countries, especially among men, may be associated with a Westernized diet and lifestyle [1,2]. Colorectal cancer ranked top in cancer cases in 16 countries, including numerous Eastern European countries, indicating a severe epidemiological issue. Most countries have adopted screening programs, which enable early detection of CRC. Despite efficient screening programs, premature mortality has a significant influence on years of life lost (YLL) [1]. According to Sun et al. [2], the SDG 3.4 targets (UN Sustainable Development Goal) for CRC may be challenging to meet by 2030, as lowering premature death necessitates complex and costly strategies such as widespread screening programs, early diagnosis, and effective treatment. Countries with well-developed prevention programs and low mortality rates may struggle to lower the premature deaths by another one-third, despite a notable downward trend. The potential gain in life expectancy (PGLE) suggests that eradicating or successfully treating CRC could greatly increase life expectancy in the general population [1]. These findings underscore the importance of expanding CRC prevention and early diagnosis, particularly in industrialized nations, to minimize premature deaths and enhance life expectancy. As a result, it is crucial to investigate innovative procedures that can quickly and efficiently enhance current diagnostics.

Relaxin-2 (RLN2) is a hormone initially produced as a single-chain pre-prohormone in males by the prostate and females by the corpus luteum. It belongs to a family of peptides that also comprises Relaxin-1 and Relaxin-3 and possesses insulin-like activity, with approximately 25% structural homology with insulin [3]. RLN2 has been discovered to play important roles in various physiological processes, including implantation of embryo into uterine wall, endometrial cell vascularization and differentiation [4], improvement of sperm motility within the male reproductive tract [5], induction of collagen breakdown [6], increased vascularization and renal function in pregnancy [7], or hemodynamic regulation in patients experiencing acute heart failure [8]. One of these mechanisms is RLN2-induced NO production, which has been found to be involved in oncogenic cell migration and growth. In breast cancer cells such as MCF-7, RLN2 has been primarily shown to induce NO production by upregulating inducible nitric oxide synthase (iNOS), possibly to promote increased blood flow and angiogenesis in these cells [9,10]. RLN2 overexpression has also been reported in other neoplasms such as prostate, thyroid, and endometrial cancers [11,12,13]. Elevated circulating levels of RLN2 were also found in patients with breast cancer [14]. Notably, interference with RLN2 expression in vitro reduced metastasis and promoted prostate adenocarcinoma cell death, highlighting its potential as a therapeutic target. Certainly, inhibition of RLN2 and/or its receptor RXFP1 in prostate cancer cells has been shown to inhibit metastasis and invasiveness in vitro [15]. More recent reports indicate that RLN2 plays a complex, bidirectional role in cancer, exhibiting both pro- and antitumor effects depending on the tumor type [16]. In breast cancer, RLN2 produced by tumor-infiltrating neutrophils (TANs) has been shown to stimulate tumor cell migration by activating the G-CSF-RLN2-MMP-9 axis, leading to increased invasiveness and metastatic potential [17]. RLN2 may also modulate pathways related to extracellular matrix remodeling, angiogenesis, and apoptosis, influencing tumor growth dynamics and metastatic potential [18]. In some cancers, such as prostate cancer, RLN2 has been shown to activate the Wnt pathway, promoting tumor cell proliferation and survival [19]. On the other hand, there are reports of potential tumor-suppressing effects of RLN2, emphasizing the need for further investigation into its role as a biomarker and potential therapeutic target [16].

What is more, data on RLN2 in colorectal cancer (CRC) remain limited. However, experimental evidence suggests that RLN2 plays a significant role in the stroma-rich tumor microenvironment. RLN2 exhibits potent antifibrotic properties, and, by binding to its receptor RXFP1 (LGR7), it stimulates the expression of matrix metalloproteinases (MMPs), leading to the degradation of collagen and other extracellular matrix components. Consequently, RLN2 facilitates the infiltration of immune cells, including T lymphocytes, into the tumor, thereby disrupting the stromal barrier that impairs the effectiveness of immunotherapy [20,21]. In animal models of CRC, CAR-T with lymphocytes engineered to secrete RLN2 demonstrated enhanced tumor infiltration and superior antitumor activity compared with conventional CAR-T cells [20]. Moreover, RLN2 gene therapy combined with chemotherapy (FOLFOX) and IL-12 induced robust immune memory responses and prolonged survival in a model of CRC liver metastases [21]. These effects are thought to result from both modification of the tumor microenvironment (stromal inactivation and facilitation of effector cell migration) and indirect enhancement of immunological and cytotoxic therapies. Experimental evidence suggests that RLN2 may influence key processes in colorectal cancer, including extracellular matrix remodeling, angiogenesis, and immune cell infiltration.

YKL-40 or Chitinase-3-like Protein 1 (CHI3L1) is a 40-kDa glycoprotein secreted by various cells such as macrophages, chondrocytes, neutrophils, and synovial fibroblasts, and binds heparin and chitin in a non-enzymatic manner [22,23]. It plays a significant role in carcinogenesis by amplifying the inflammatory response, which promotes tumor progression. YKL-40 promotes a tumor-promoting environment by, among others, stimulating the production of inflammatory mediators that support tumor growth, angiogenesis, and metastatic potential. Chronic inflammation induced by YKL-40 may also facilitate tumor cell migration and invasion, thus contributing to tumor progression and spread [24]. Its increased concentration has been observed in diseases such as Alzheimer’s disease, COPD, asthma, osteoarthritis, acute kidney injury, inflammatory bowel disease, and cancers, including cervical, endometrial, and esophageal [23,25,26,27,28,29,30,31,32]. YKL-40 is involved in multiple key signaling pathways that promote growth, invasion, angiogenesis, and immunosuppression in cancer. Interestingly, overexpression of YKL-40 has been shown to increase the activity of PI3K, AKT, mTOR, and their phosphorylated forms, while silencing YKL-40 decreases the activity of these proteins in melanoma [33]. Furthermore, YKL-40 has been shown to stimulate the ERK1/2 pathway by activating CD138 receptors and αvβ3 integrin, promoting angiogenesis, proliferation, and invasion of cancer cells in cutaneous T-cell lymphoma [34]. Additionally, in endometrial cancer, YKL-40 has been shown to regulate angiogenesis by influencing VEGF expression and VEGFR2 activation [35]. High expression of YKL-40 was also detected in the HCT116 and Caco2 lines, where it correlated with the activation of EMT markers and the severity of metastatic features of CRC [36].

Therefore, the primary aim of this study was to evaluate the diagnostic usefulness of serum RLN2 and YKL-40 as biomarkers in patients with colorectal cancer, compared to conventional ones (CEA and CA19-9), while the secondary aim was to explore their associations with tumor stage and therapy. We hypothesized that those parameters would demonstrate significant diagnostic utility in CRC detection and monitoring.

## 2. Results

Table 1 compares the serum levels of Relaxin-2 (RLN2), Chitinase-3-like protein 1 (CHI3L1/YKL-40), CEA, and CA 19-9 in colorectal cancer patients and a control group. The non-parametric Mann–Whitney U test revealed that the overall cancer group had significantly higher levels of YKL-40, CEA, and CA 19-9 compared to the healthy group (*p* < 0.05).

Continuing our analyses using the Mann–Whitney U test, we checked whether there were significant differences between patient groups based on cancer type (colon/rectal), and whether there were differences in concentrations in patients who were referred for additional radiotherapy or chemotherapy after blood sampling and before surgery. We observed no statistically significant differences between cancer types. Interestingly, in the case of preoperative treatment, YKL-40 concentration was significantly higher in patients referred for additional radiotherapy or chemotherapy (Me: 65,537.60 pg/mL) compared to patients who were referred directly for surgery (Me: 42,033.02 pg/mL; *p* = 0.035).

Furthermore, we carried out more specific analysis with Kruskal–Wallis and post-hoc tests following the division of the whole CRC group into two groups according to cancer progression (without—TNM I–II and with—TNM III–IV metastases). Kruskal–Wallis analysis showed significant results for all the parameters under study (Table 2). However, based on the received post-hoc results, it is probable that only YKL-40 and CEA levels are associated with tumor growth, and RLN2 and CA 19-9 are associated only with advanced stages of cancer, when metastases occur. Additionally, box-and-whisker plots for each parameter at each stage of advancement were added as Supplementary Materials (Supplementary Figure S1).

As with the previous test, we performed additional analyses, checking multiple independent samples for statistically significant differences in histological types (adenocarcinoma/mucinous adenocarcinoma/squamous cell carcinoma) and histological grade (G feature). Nevertheless, we did not observe any significant differences between concentrations of all parameters in selected groups.

Table 3 summarizes the diagnostic performance of all analyzed parameters, including sensitivity (SE), specificity (SP), accuracy (ACC), predictive values (PPV, NPV), and the area under the ROC curve (AUC). We showed that higher SE from newly tested parameters revealed YKL-40 (65%); however, it was lower than the SE of the routine marker, CEA, but higher than CA 19-9 (50%), which was the lowest of all SE results. In case of SP, RLN2 and YKL-40 showed high values (76.90% and 77.50%, respectively), which were lower than SE of CA 19-9 (80%), but higher than CEA (72.50%). The obtained accuracy was high for RLN2 and YKL-40 (62.20% and 69.20%, respectively): higher than ACC of CA 19-9 (60%), but lower than CEA (85.80%). Positive predictive values were high in all tested parameters (from 83.00–87.10%). Negative predictive value was calculated at 52.50% for YKL-40 and 45.50% for RLN2, which was higher than for CA 19-9 but lower than for CEA.

**Figure 1:**
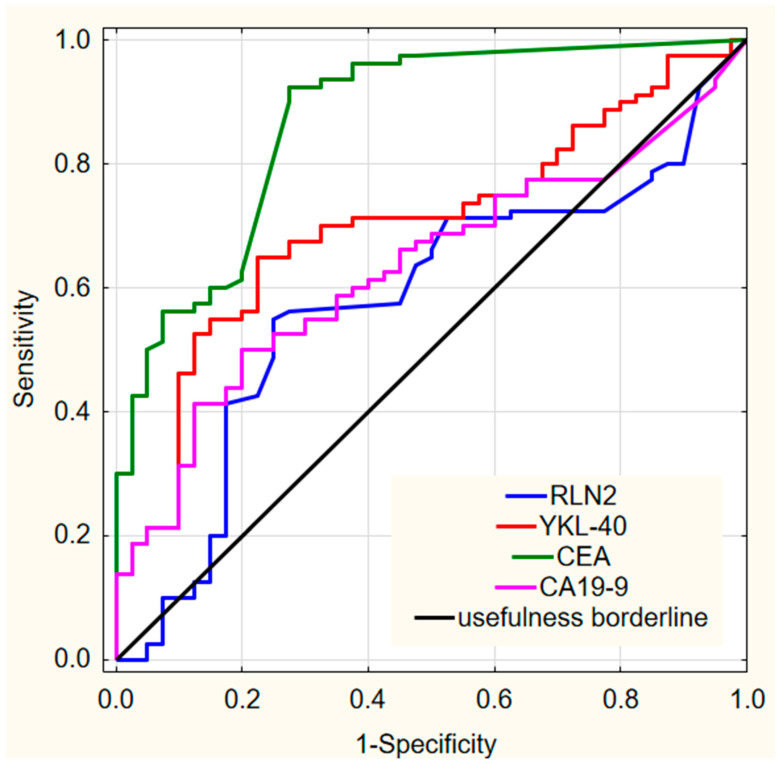
Receiver operating characteristics for all ROC analysis results. Abbreviations: RLN2—Relaxin-2; YKL-40—Chitinase-3-like protein 1; CEA—Carcinoembryonic Antigen; CA 19-9—Carbohydrate Antigen 19-9.

We found that, among all tested parameters, YKL-40 showed the highest area under the ROC curve (AUC = 0.702; *p* < 0.001) in the total colorectal cancer group, although it remained lower than the AUC obtained for CEA (AUC = 0.869; *p* < 0.001). Notably, the newly evaluated marker demonstrated a higher AUC value compared with CA 19-9 (AUC = 0.632; *p* = 0.009). In contrast, the AUC for RLN2 did not reach statistical significance. The graphical representation of all ROC analyses is provided in Figure 1. The AUCs for all parameters shown in Figure 1 were significantly greater than 0.5, indicating diagnostic relevance (*p* < 0.05 for all comparisons).

**Figure 2:**
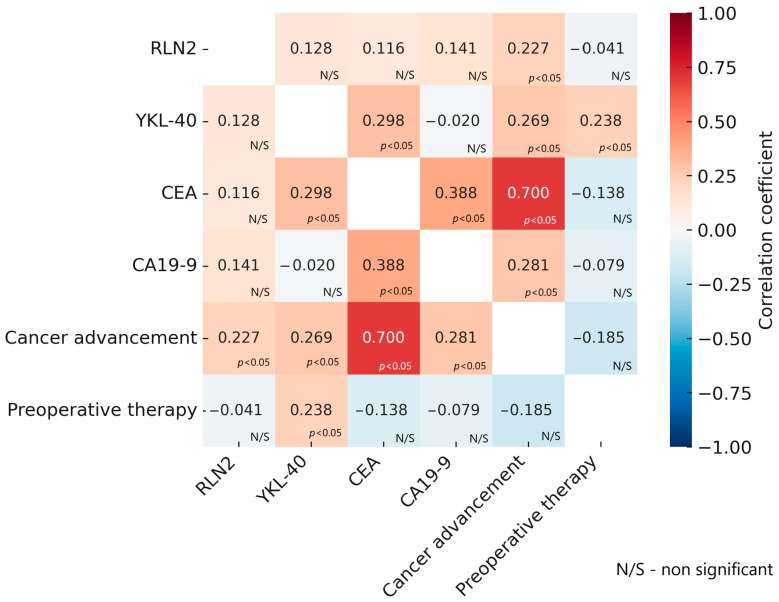
Spearman’s rank correlation coefficient for the tested variables. Abbreviations: RLN2—Relaxin-2; YKL-40—Chitinase-3-like protein 1; CEA—Carcinoembryonic Antigen; CA 19-9—Carbohydrate Antigen 19-9.

To complement the statistical analysis, Spearman’s rank correlation coefficient was calculated to assess the strength and direction of the monotonic relationship between variables. The obtained results are shown in Figure 2. 

Correlation analysis between the studied biomarkers and clinical parameters revealed that the strongest positive association was between CEA concentration and disease stage, indicating that CEA levels significantly increase with colorectal cancer progression. CEA also showed a weak positive correlation with CA 19-9 and YKL-40. Both YKL-40 and CA19-9, as well as RLN2, correlated weakly but positively with cancer stage, suggesting their partial usefulness as indicators of progression. Analysis of the relationship with preoperative therapy showed a weak positive correlation with YKL-40, which confirms the previous observation that the concentration of YKL-40 was significantly different in groups of patients who were or were not referred for additional therapy before surgery.

## 3. Discussion

Early detection of cancer and screening diagnostics play a key role in improving patient prognosis. Systematic screening allows for the detection of changes at the stage where treatment is most effective, significantly increasing the chances of a full recovery. Although CRC screening diagnostics have significantly improved disease detection, current methods remain insufficient, and many cases are diagnosed only at an advanced stage. That is why it is so important to search for new parameters that would indicate an ongoing cancer process.

To date, no studies have been published assessing serum RLN2 levels in patients with colorectal cancer, making our work the first clinical serum-based evaluation of RLN2 in CRC and a unique contribution to the development of knowledge about biomarkers for this disease. However, elevated RLN2 levels have been reported in other cancers, such as prostate cancer, breast cancer, and osteosarcoma, where they correlate with disease progression and poorer prognosis [37,38], which prompted us to conduct research on this parameter. It has also been shown that RLN2 may play a role in angiogenesis and extracellular matrix remodeling, which may be important in tumor progression. In our study, serum RLN2 levels in patients with colorectal cancer did not show statistically significant differences compared to the control group, indicating that circulating RLN2 has limited diagnostic value in CRC. However, the observed differences in median values suggest a possible elevation of this parameter, similar to what has been reported in other cancers, such as prostate, breast, ovarian, and osteosarcoma, where higher RLN2 levels were associated with advanced disease and poorer prognosis [37,38,39]. One possible explanation for the lack of statistical significance is the limited sample size, which could have influenced the statistical power of the analysis and prevented the detection of subtle differences. Only in advanced stages has the significance been discovered. Furthermore, correlation analysis revealed only a weak relationship between RLN2 levels and tumor stage, suggesting that if RLN2 contributes to colorectal cancer progression, its effect is likely minor or indirect and mediated through other biological pathways. Nevertheless, because this is the first study assessing serum RLN2 in CRC, further research, particularly in larger and more diverse cohorts, is required to fully clarify its potential biological and clinical relevance.

We can hypothesize that although preclinical studies indicate that RLN2 may actively influence the tumor microenvironment through angiogenesis, ECM remodeling, and modulation of stromal cells, these mechanisms are primarily localized processes. This may explain why systemic RLN2 levels do not necessarily reflect intratumoral activity, resulting in the lack of diagnostic significance observed in our cohort. It is therefore plausible that RLN2 acts locally within the tumor niche while remaining below the detection threshold in peripheral circulation. Future studies incorporating tissue-level analyses or paired tumor–serum assessments would be valuable for clarifying this discrepancy.

The first mention of the usefulness of YKL-40 testing in patients with colorectal cancer appeared over 25 years ago, when Cintin et al. [40] demonstrated that patients with high preoperative serum YKL-40 concentration had significantly shorter survival. Subsequent reports on the usefulness of this parameter appeared almost 15 years later, and they assessed its usefulness as a protein that could be used for earlier detection of CRC. In the studies by Vock et al. [41] and Tarpgaard et al. [42], the authors assessed the usefulness of this parameter in metastatic colorectal cancer. They indicated that the concentrations of this parameter in cancer patients were higher compared to healthy individuals, which confirms our results not only in the entire study group but also in advanced-stage CRC associated with the presence of distant metastases. The authors confirmed earlier reports that elevated pretreatment YKL-40 was an independent biomarker of short overall survival and demonstrated that it demonstrated decreased sensitivity compared to CEA, similar to our study. Interestingly, the study by Fuksiewicz et al. [43], which included only patients with early-stage rectal cancer, demonstrated similar correlations with concentrations. However, the demonstrated diagnostic usefulness of this parameter was higher than that of CEA (AUC 0.769 vs. 0.728, respectively). The discrepancies between our findings and this study may be partially attributable to differences in study populations. In the compared study, only rectal cancer patients were included, whereas our cohort comprised both colon and rectal cancers. To explore this further, we conducted an additional subgroup analysis within our dataset. Although no statistically significant differences in biomarker levels were observed between colon and rectal cancer cases, the relatively small numbers in each subgroup limit the ability to detect subtle location-related effects. Therefore, the tumor site cannot be entirely ruled out as a contributing factor to inter-study variability. In the work of Eldaly et al. [44], it was shown that the usefulness of determining this parameter is very high and reaches AUC = 0.97, specificity = 91.7%, and sensitivity = 96%, which exceeds our results; however, the work was performed using serum from 60 patients and 12 healthy individuals, which may significantly affect the uncertainty of the presented results. Similar conclusions (but lower utility) with a similar number of patients were also found by Liu et al. [45] and Ye et al. [46]. There are also studies conducted on a larger study group, where the results were similar to ours. In the study by Johansen et al. [47], the authors demonstrated significant differences between the study groups (highest in cancer), and the determined AUC was 0.68, lower than that obtained for CEA (0.75), which is also consistent with our results. Similar results were also presented in a meta-analysis conducted by Wang et al. [48].

We also found studies assessing YKL-40 levels in patients whose blood was collected after therapy. Hermunen et al. [49] demonstrated that in patients with radically treated CRC (stages II-IV) who underwent adjuvant 5-FU-based chemotherapy, postoperatively elevated CEA in combination with CA19-9 or YKL-40 or a normal CEA associated with an elevated YKL-40 may suggest a high risk of relapse. Although the aim of our study was not to evaluate the results after therapy, these data provide additional knowledge about the significant usefulness of YKL-40 in CRC. Another study demonstrated that transient increases (≥10% increase followed by a decrease) in YKL-40 tended to be associated with favorable survival [50]. Other studies have also evaluated the usefulness of YKL-40 based on immunohistochemistry. The authors revealed that patients with I-YKL-40 expression had shorter overall and recurrence-free survival in CRCs with a high immunoscore. Their findings showed that I-YKL-40 expression was substantially associated with tumor differentiation and that I-YKL-40 expression can help with the prognosis of CRC patients [51]. There is also a work by De Robertis et al. [36] where the authors, using cell lines, also confirmed that high-YKL-40-expressing cells showed increased motility, invasion, and proliferation, and additionally tested serum samples from CRC patients had elevated YKL-40 levels, which simultaneously correlated with high-grade tumors, which clearly confirms previous reports and our findings.

Taken together, the available evidence shows that YKL-40 has been consistently identified as a marker associated with colorectal cancer across diverse study designs, populations, and methodological approaches. Early studies highlighted its prognostic relevance, while later work confirmed elevated levels in both early-stage and metastatic disease, although the magnitude of diagnostic accuracy varied substantially. Divergent findings, particularly those reporting exceptionally high AUC values, appear largely attributable to small sample sizes and heterogeneous study designs. Larger cohorts and meta-analytic data demonstrate a more moderate but reproducible diagnostic performance, which aligns closely with our results. Collectively, these studies suggest that YKL-40 is not a standalone superior biomarker, but rather a biologically meaningful molecule with stable, complementary value alongside established markers, such as CEA. Our findings, therefore, fit within and help refine the broader evidence base by providing additional support for the moderate yet consistent diagnostic utility of YKL-40 in CRC.

Despite the promising findings, several limitations of this study should be acknowledged. The use of the same dataset to define biomarker cut-offs and assess diagnostic performance may overestimate accuracy. The total sample included 80 patients and 40 healthy controls, which restricts statistical power, particularly for subgroup analyses (e.g., only one patient with squamous cell carcinoma). This modest sample size may limit the generalizability of the results and could affect the observed trends, especially for biomarkers such as RLN2, where non-significant results might reflect underpower rather than a lack of biological relevance. Additionally, the study population was relatively homogeneous in terms of demographic and clinical characteristics, which may influence serum biomarker concentrations. Future studies with larger, multicenter, and more diverse cohorts are warranted to confirm these findings, validate the diagnostic utility of YKL-40 and RLN2, and explore their potential integration into multi-marker panels for colorectal cancer detection and monitoring. Although a formal multi-marker panel analysis was not performed in the present study, our results suggest that combining YKL-40 with conventional biomarkers such as CEA could enhance diagnostic performance. YKL-40 exhibited moderate sensitivity (65%) and high specificity (77.5%), while CEA showed higher sensitivity but lower specificity. These complementary characteristics indicate that a combined approach may improve overall accuracy for detecting colorectal cancer, particularly in cases where a single marker provides ambiguous results. Future studies should focus on evaluating multi-marker panels and integrating YKL-40 with other serum biomarkers to confirm and optimize its clinical utility. Although the present study suggests that YKL-40 may complement established markers such as CEA, the dataset was not sufficiently powered to construct a statistically reliable multivariate model or to calculate a combined panel AUC. Therefore, the concept of a CEA/YKL-40 diagnostic panel remains exploratory.

## 4. Materials and Methods

### 4.1. Patients

A total of 80 patients with colorectal cancer (CRC) diagnosed by the oncology team were enrolled in the study between 2019 and 2023 (Table 4). All participants received treatment at the Department of Oncological Surgery and the Specialized Cancer Treatment Units of the Maria Sklodowska–Curie Oncology Center in Bialystok, Poland. Tumors were staged and classified using the UICC-TNM classification (International Union Against Cancer Tumor-Node-Metastasis).

Histopathological examination of CRC was accomplished by microscopic examination of tissue specimens. The TNM staging system was applied to group all patients, which comprises the stage of the tumor, depth of invasion (T factor), lymph node involvement (N factor), presence of distant metastasis (M factor), and histological grade (G factor). Pretreatment staging consisted of physical examination, laboratory blood tests, and computed tomography (CT). In patients with rectal cancer, pelvic MRI was also performed. The performance status in all patients was assessed using the ECOG—Eastern Cooperative Oncology Group performance score.

The control group consisted of 40 healthy individuals with no history of cancer or other diseases of the abdominal organs who had a negative colonoscopy. Basic biochemical and hematological tests were performed to confirm health status. Exclusion criteria for this group included a history of cancer in the past, pregnancy, obesity, current infections, and chronic respiratory, gastrointestinal, or systemic illness (e.g., systemic lupus erythematosus, rheumatoid arthritis).

### 4.2. Biochemical Analyses

Venous blood samples were obtained from all participants prior to the initiation of any therapeutic procedures (surgical, chemotherapeutic, or radiotherapeutic). Samples were collected into clot activator tubes (S-Monovette, SARSTEDT, Sarstedt, Germany), centrifuged to separate serum, and stored at −80 °C until analysis. The concentrations of YKL-40 and RLN2 were measured using a customized Luminex Discovery Assay (LXSAHM, Luminex Human Discovery Assay, R&D Systems, Abingdon, UK). The assay was using Human Standard Cocktail L (catalog no. 894863, magnetic bead region 56) for RLN2 and Human Standard Cocktail C (catalog no. 894368, magnetic bead region 20) for YKL-40. Serum concentrations of conventional tumor markers were measured with a chemiluminescent microparticle immunoassay (CMIA) (Abbott, Chicago, IL, USA) following the manufacturer’s protocol. All standards, controls, and samples in the Luminex assay were analyzed in duplicate according to the manufacturer’s instructions.

### 4.3. Statistical Analysis

The statistical analysis was achieved using TIBCO Statistica 14.1.0.4. The initial statistical Shapiro-Wilk test indicated that all parameter levels were not normally distributed. Accordingly, statistical analysis was completed by non-parametric tests (Mann–Whitney U test, Kruskal–Wallis test, and by multivariate analysis of data through the post-hoc test). Diagnostic sensitivity (SE), specificity (SP), accuracy (ACC), and positive and negative predictive values for test results (PPV and NPV, respectively) were calculated using cut-off values predicted with the use of the Youden’s index, and each of the investigated parameters were as follows: Relaxin-2—80.81 pg/mL; YKL-40—39,891.51 pg/mL; CEA—1.45 ng/mL; CA 19-9—5.03 U/mL.

Additionally, we checked whether there were any correlations between the studied parameters using Spearman’s rank correlation test. For each parameter, we also enrolled a receiver-operating characteristic (ROC) curve for assessment of diagnostic accuracy. A *p*-value of less than 0.05 was considered statistically significant.

## 5. Conclusions

Novel, robust, and minimally invasive biomarkers are critically needed to improve early identification and risk stratification of colorectal cancer (CRC), as current screening tests and conventional markers such as CEA and CA 19-9 have low sensitivity and specificity, particularly for early conditions. RLN2 is a novel candidate, and preclinical studies show that it can impact the tumor microenvironment, immune cell infiltration, and extracellular matrix remodeling, potentially increasing the efficacy of immunotherapy. However, our clinical data revealed no statistically significant differences in RLN2 serum levels between CRC patients and controls, and it only demonstrated a weak connection with tumor stage, indicating that its diagnostic value is currently limited. In contrast, YKL-40 is consistently elevated in CRC patients, corresponds with advanced stage and poor differentiation, and is considered a powerful prognostic marker for survival and recurrence. While YKL-40 does not outperform CEA in sensitivity, it does increase diagnostic accuracy when paired with known markers. Elevated YKL-40 levels can also help identify patients who are at a higher risk of recurrence or have not responded well to treatment. The addition of YKL-40 to biomarker panels may improve early identification and customized surveillance in CRC. Future studies with larger, prospective cohorts are warranted to validate the potential of a CEA/YKL-40 biomarker panel, particularly for early-stage colorectal cancer detection and monitoring of disease progression.

## Figures and Tables

**Table 1:** Levels of RLN2, YKL-40, CEA, and CA 19-9 in CRC and control patients’ serum.

Parameter		Colorectal Cancer	Control Group	*p* *
RLN2 [pg/mL]	Median value	81.35	61.75	0.103
	Minimum–Maximum	6.09–522.20	6.09–902.35	
	Q1–Q3	46.93–130.68	49.68–80.51	
YKL-40 [pg/mL]	Median value	47,695.45	31,274.02	<0.001
	Minimum–Maximum	6085.85–828,508.00	3287.52–95,870.72	
	Q1–Q3	26,726.20–73,836.90	19,160.50–39,215.77	
CEA [ng/mL]	Median value	2.82	0.50	<0.001
	Minimum–Maximum	0.50–1844.00	0.50–7.82	
	Q1–Q3	1.73–11.02	0.50–1.73	
CA 19-9 [U/mL]	Median value	4.91	3.31	0.018
	Minimum–Maximum	2.00–1357.00	2.00–39.29	
	Q1–Q3	2.42–11.91	2.09–4.78	

* *p*-value obtained in Mann–Whitney U test; Abbreviations: RLN2—Relaxin-2; YKL-40—Chitinase-3-like protein 1; CEA—Carcinoembryonic Antigen; CA 19-9—Carbohydrate Antigen 19-9; Q1–Q3—first quartile—third quartile (interquartile range, IQR).

**Table 2:** Kruskal–Wallis and post-hoc test analysis results.

Parameter		RLN2	YKL-40	CEA	CA 19-9
Kruskal–Wallis *p*-value		0.031	0.002	<0.001	0.008
Post-hoc *p*-value	Control vs. I + II	1.000	0.003	<0.001	0.936
	Control vs. III + IV	0.043	0.010	<0.001	0.007
	I + II vs. III + IV	0.115	1.000	<0.001	0.122

Abbreviations: RLN2—Relaxin-2; YKL-40—Chitinase-3-like protein 1; CEA—Carcinoembryonic Antigen; CA 19-9—Carbohydrate Antigen 19-9.

**Table 3:** Diagnostic criteria of tested parameters in CRC patients.

Parameters	Diagnostic Criteria	CRC
RLN2	SE	55.00%
	SP	76.90%
	ACC	62.20%
	PPV	83.00%
	NPV	45.50%
	AUC	0.593 (*p* = 0.090)
YKL-40	SE	65.00%
	SP	77.50%
	ACC	69.20%
	PPV	85.20%
	NPV	52.50%
	AUC	0.702 (*p* < 0.001)
CEA	SE	92.50%
	SP	72.50%
	ACC	85.80%
	PPV	87.10%
	NPV	82.90%
	AUC	0.869 (*p* < 0.001)
CA 19-9	SE	50.00%
	SP	80.00%
	ACC	60.00%
	PPV	83.30%
	NPV	44.40%
	AUC	0.632 (*p* = 0.009)

Abbreviations: RLN2—Relaxin-2; YKL-40—Chitinase-3-like protein 1; CEA—Carcinoembryonic Antigen; CA 19-9—Carbohydrate Antigen 19-9; CRC—Colorectal Cancer; SE—Sensitivity; SP—Specificity; ACC—Accuracy; PPV—Positive Predictive Value; NPV—Negative Predictive Value; AUC—Area Under the Curve.

**Table 4:** Characteristics of colorectal cancer (CRC) and healthy control groups.

Group Studied	Patient Characteristics	Number of Patients
Colorectal Cancer		80
	Gender:	
	Female	28
	Male	52
	Type:	
	Colon Cancer	33
	Rectal Cancer	47
	Histological subtype:	
	Adenocarcinoma	71
	Mucinous adenocarcinoma	8
	Squamous cell carcinoma	1
	TNM Stage:	
	I	20
	II	20
	III	20
	IV	20
	Depth of tumor invasion:	
	T1	4
	T2	19
	T3	45
	T4	12
	Nodal involvement:	
	N0	45
	N1	23
	N2	12
	Distant metastasis:	
	M0	60
	M1	20
	Histological grade:	
	G1	6
	G2	65
	G3	9
	Preoperative therapy:	
	Yes	27
	No	53
	Age:	32–89
Control Group		40
	Gender:	
	Female	22
	Male	18
	Age:	22–75

## Data Availability

The data presented in this study are available on request from the corresponding author. Key data are stated in the text.

## Supplementary Materials

The following supporting information can be downloaded at: https://www.mdpi.com/article/10.3390/ijms262311601/s1.
