# Supplementary figures and images for: Serum YKL-40, but Not Relaxin-2, Shows Diagnostic Utility as an Adjunct Biomarker in Colorectal Cancer
Source: Int J Mol Sci. 2025 Nov 29;26(23):11601. doi: 10.3390/ijms262311601 (PMC12691882; doi:10.3390/ijms262311601)

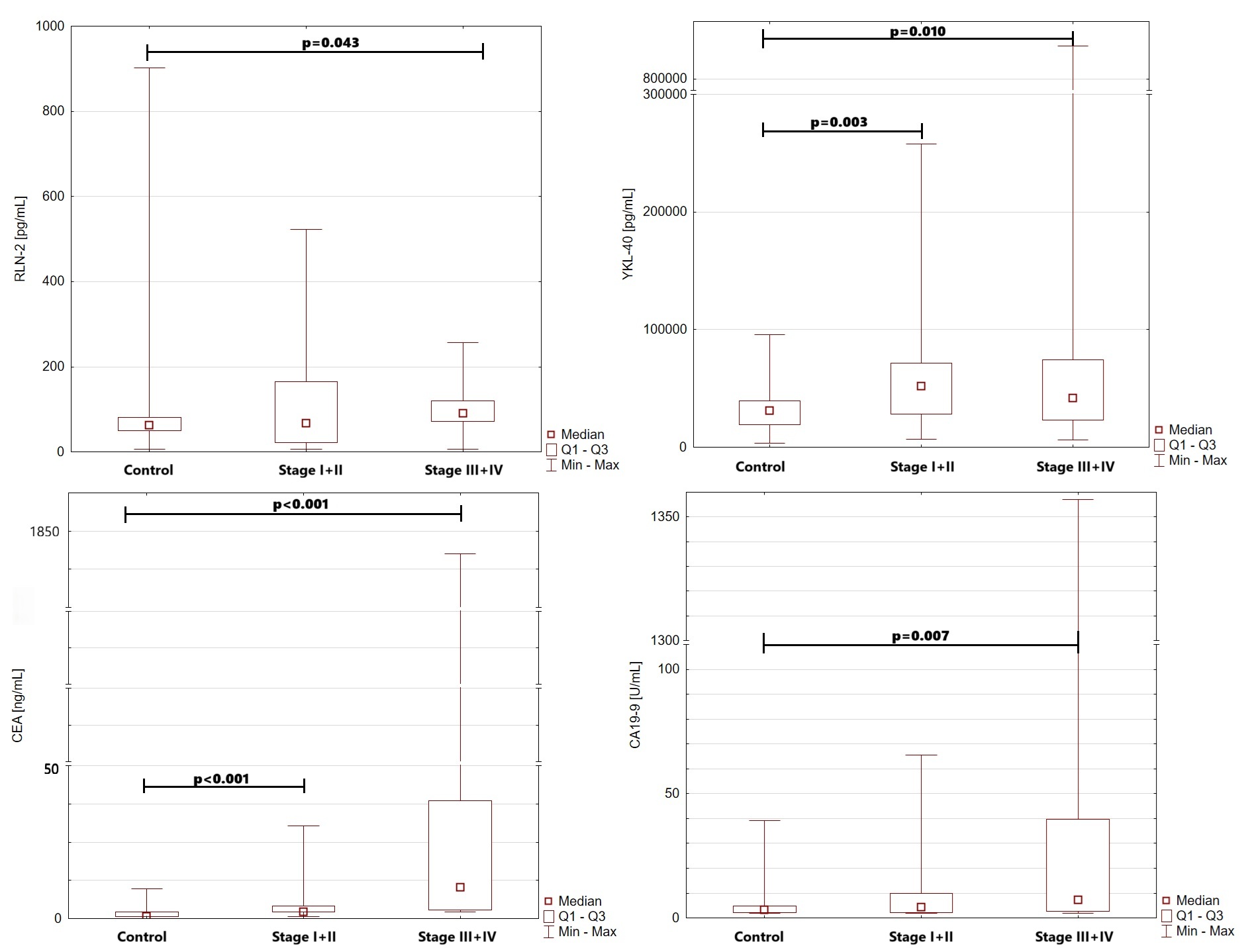

Supplement: Supplementary file 1 [file ijms-26-11601-s001.zip › ijms-3994998-supplementary.jpg]
